# Contralateral Acute Subdural Hematoma Immediately After Irrigation of a Chronic Subdural Hematoma: A Case Report

**DOI:** 10.7759/cureus.97144

**Published:** 2025-11-18

**Authors:** Ryo Matsuzaki, Yutaka Fuchinoue, Yuki Sakaeyama, Shuhei Kubota, Nobuo Sugo

**Affiliations:** 1 Neurosurgery, Toho University, Tokyo, JPN

**Keywords:** acute subdural hematoma, anticoagulant, chronic subdural hematoma, middle cerebral artery infarction, remote acute subdural hematoma

## Abstract

Few reports describe contralateral subdural hematoma (SDH) after chronic SDH (CSDH) surgery. In this article, we present a rare case of postoperative complication. The patient was a 76-year-old woman with a history of cerebral infarction due to atrial fibrillation and who was taking an anticoagulant. She presented with a disturbance of consciousness on the Glasgow Coma Scale (GCS) E2V5M6. Computerized tomography (CT) revealed a left CSDH; drainage was performed immediately. After surgery, the CT scan revealed an acute SDH (ASDH) on the contralateral side. Three hours later, the hematoma increased, and she became comatose with a GCS of 1V1M1, and surgery was performed. The speculated cause of the contralateral hemorrhage was the damage to the bridging veins and superficial cerebral vessels due to a sudden change in intracranial pressure, combined with brain fragility and a prolonged prothrombin time (PT) and international normalized ratio (INR) resulting from warfarin therapy. Caution during the postoperative period and follow-up is required for early identification of these cases.

## Introduction

Chronic subdural hematoma (CSDH) is a common neurosurgical condition typically managed with burr-hole irrigation and drainage, whereas acute SDH (ASDH) often results from traumatic bleeding and usually requires prompt surgical intervention. Although postoperative bleeding after CSDH surgery usually occurs on the ipsilateral side, fatal complications such as intracranial hemorrhage may occasionally arise. In rare cases, contralateral or remote SDHs, intracerebral hemorrhages, and subarachnoid hemorrhages have also been reported [1,2,3]. Contralateral ASDH is exceptionally rare, but early recognition is critical because neurological deterioration can develop rapidly within hours, as noted in previous case reports [1,2]. We report a case of contralateral ASDH that occurred immediately after irrigation of a CSDH, describing its presentation, imaging timeline, hypothesized mechanisms, and perioperative anticoagulation considerations.

## Case presentation

A 76-year-old woman was admitted to the emergency room with impaired consciousness. She had sustained a minor head injury a few months before presentation and had a medical history of cardiogenic cerebral embolism in the right middle cerebral artery territory, for which she was taking warfarin. On arrival, her Glasgow Coma Scale (GCS) score was E2V5M6, and she presented with right hemiparesis. Vital signs and general examination findings were normal. Laboratory tests revealed unexplained thrombocytopenia, with a platelet count of 84,000/μl and a prolonged patient’s prothrombin time (PT) and international normalized ratio (INR) of 1.7.

**Figure 1:**
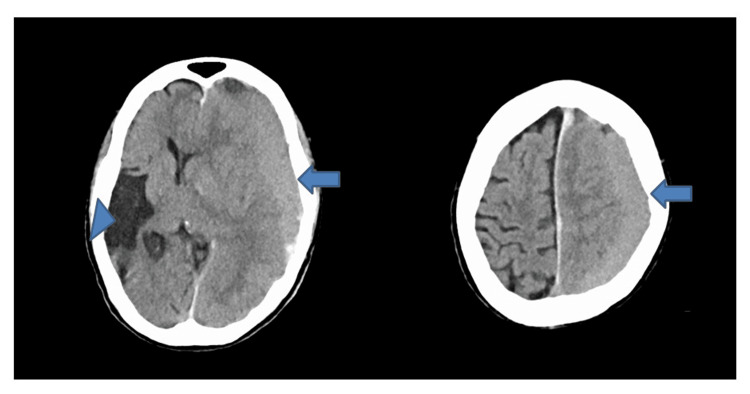
An initial head CT obtained 10 minutes after arrival showed a left chronic subdural hematoma (20 mm thick) (arrow) with a 15 mm midline shift to the right and a hypodense area occupying the right temporoparietal region (arrowhead), with no contralateral hematoma.

A head CT scan obtained 10 minutes after arrival showed a left CSDH measuring 20 mm in maximal thickness with a 15 mm midline shift to the right, as well as a hypodense area in the right temporoparietal region (Figure 1). Since postoperative bleeding is generally rare after CSDH surgery, the patient was considered to have a low risk of hemorrhage but a high risk of thrombosis due to a CHADS₂ score of 5. According to the European Society of Cardiology (ESC) guidelines, an INR below 2.0 is considered acceptable for minor bleeding events [3], so we performed the surgery without reversing warfarin.

**Figure 2:**
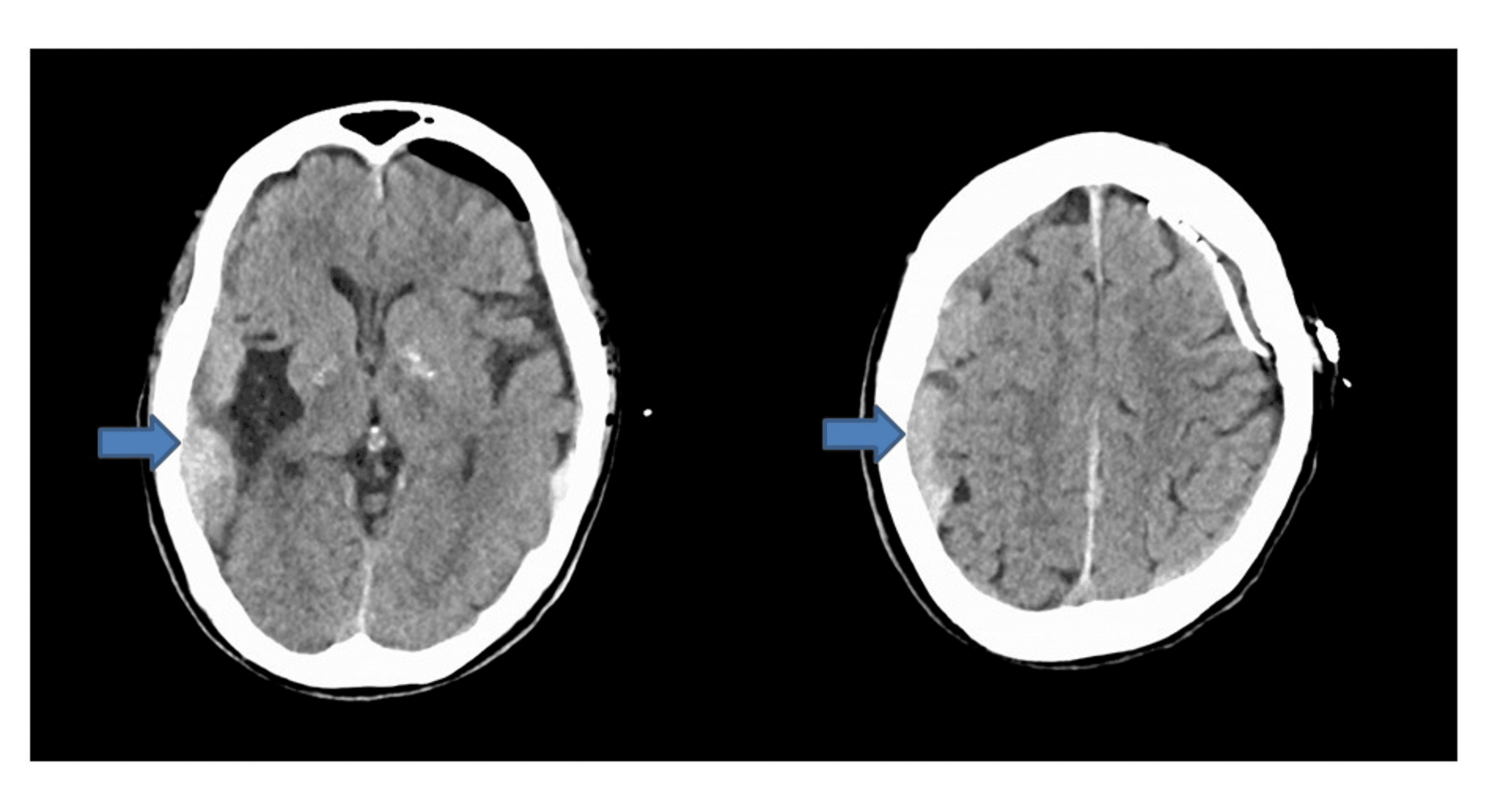
Immediate postoperative CT (six hours and 30 minutes after arrival) following the first surgery, demonstrated complete removal of the left hematoma, no midline shift, and the appearance of a new right acute subdural hematoma (22 mm thick) (arrow).

**Figure 3:**
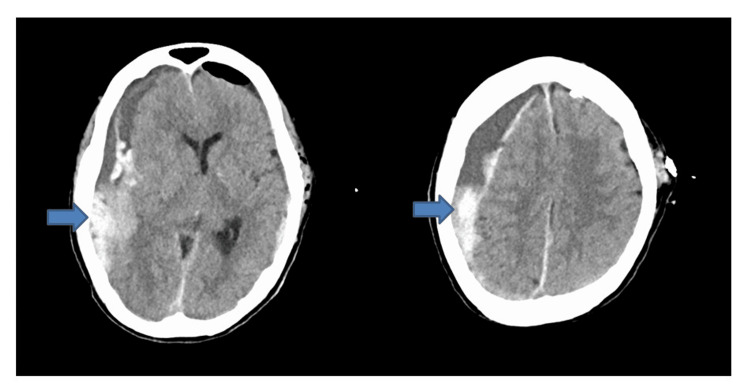
Follow-up CT performed nine hours and 27 minutes after arrival showed further enlargement of the right hematoma (30 mm thick) (arrow) with a 13 mm midline shift to the left, while the left side remained clear.

The first surgery was performed five hours after arrival. The drainage fluid revealed a dark red hematoma. The immediate postoperative CT, obtained six hours and 30 minutes after arrival, showed complete removal of the left hematoma and no midline shift but revealed a new right ASDH measuring 22 mm in thickness (Figure 2). A follow-up CT nine hours and 27 minutes after arrival demonstrated enlargement of the right hematoma to 30 mm, with a 13 mm midline shift to the left (Figure 3).

The patient’s level of consciousness deteriorated rapidly to GCS E1V1M1, accompanied by a convulsive seizure, for which diazepam was administered. Emergency surgery was immediately performed 10 hours and 45 minutes after arrival. During incision of the dura mater through a burr hole near the hematoma, layering and separation of fluid and clot components were observed intraoperatively, consistent with acute-on-chronic bleeding. Although burr-hole surgery is not the standard treatment for ASDH, the skin incision was designed so that it could be extended to a standard craniotomy if needed. Intraoperatively, most of the hematoma was successfully evacuated, and sufficient decompression was achieved, so craniotomy was unnecessary.

**Figure 4:**
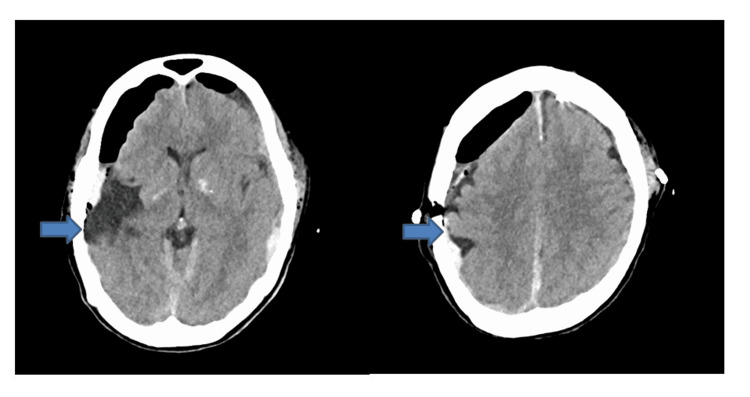
Postoperative CT obtained 12 hours after arrival (after the second surgery) confirmed complete removal of the right hematoma (arrow) and resolution of the midline shift.

The postoperative CT obtained 12 hours after arrival confirmed complete removal of the right hematoma and resolution of the midline shift (Figure 4). The patient’s postoperative course was uneventful. She was discharged after one week with no neurological deficits, corresponding to a score of 5 on the Glasgow Outcome Scale (GOS) and 0 on the modified Rankin Scale (mRS), indicating full recovery of daily activities and independence. Anticoagulant therapy was resumed after confirming stable hemostasis. One year later, she remains asymptomatic and neurologically intact.

## Discussion

An increase in contralateral hematoma after unilateral irrigation in bilateral CSDH is commonly encountered in routine clinical practice [4]. Contrastingly, postoperative contralateral hemorrhage in unilateral CSDH, as in the present case, is rare. Remote hemorrhagic events following surgeries that involve marked brain shift, such as cerebrospinal fluid drainage or removal of large space-occupying lesions, have been well documented in neurosurgical literature [1]. However, contralateral ASDH occurring immediately after CSDH evacuation remains a rare presentation [5]. Several factors could be responsible for contralateral bleeding after CSDH surgery. One such factor is the sudden change in intracranial pressure associated with the disappearance of the hematoma due to surgery. The change in intracranial pressure causes damage to the contralateral bridging vein [5]. Similar mechanisms have been described in previous reports. Shen et al. reported a case of contralateral ASDH occurring immediately after evacuation of a traumatic ASDH, attributing it to rapid decompression and brain shift leading to rupture of bridging veins [6]. Likewise, Wang et al. described postoperative parenchymal hemorrhage after burr-hole drainage for CSDH, in which sudden intracranial pressure change and cerebral hyperperfusion were considered major contributing factors [7,8]. These observations support the hypothesis that abrupt brain shift or perfusion alteration after hematoma evacuation can precipitate remote or contralateral bleeding. Other factors have been noted to decrease elastance in brain tissue after stroke [9]. In this patient, encephalomalacia following a prior insular infarction may have resulted in local anatomical changes forming a subarachnoid pouch-like space, which could have increased the susceptibility to bleeding. In addition, several systemic factors may have contributed to postoperative bleeding in this patient, including older age, thrombocytopenia, and anticoagulation therapy with warfarin. Furthermore, thrombocytopenia alone may not have directly caused postoperative bleeding, but in combination with anticoagulation therapy and other contributing factors, it could have increased the overall risk of hemorrhage.

In the present case, an ASDH developed near the site of the old infarction contralateral to the CSDH, suggesting a decrease in elastance and fragility in the brain tissue associated with cerebral infarction. The course of this case might also have been affected by the formation of a subdural hygroma from the drainage of the cerebrospinal fluid due to a rupture of the arachnoid membrane. Furthermore, a CT scan was performed three hours after surgery, and a hematoma forming a fluid level was noted in the present case. During surgery, we observed layering and separation of fluid and clot components within the hematoma cavity, consistent with the findings noted on the preceding CT scan. This suggested that cerebrospinal fluid initially drained from the ruptured arachnoid membrane and formed a subdural hygroma, which might have triggered the disruption of the superficial cerebral vessels. An et al. also noted the association between postoperative ASDH and subarachnoid hygroma [9]. Other mechanisms include oral anticoagulants, which are systemic factors, and abnormal blood coagulation capacity [10]. 

In our case, the patient had to be treated with warfarin for cardiogenic cerebral embolism. The incidence of CSDH is more than 42 times higher with oral administration of warfarin [10]. Hematologic diseases have also been reported to increase the risk of bleeding after CSDH surgery. Moreover, thrombocytopenia of an unknown cause could be involved in this case [2]. Previous reports have suggested that desmopressin administration may help reduce perioperative bleeding by enhancing platelet function in patients with thrombocytopenia or platelet dysfunction [11]. In retrospect, desmopressin administration might have been considered in this patient to further minimize the risk of postoperative hemorrhage. It is speculated that the cause of contralateral hemorrhage is the damage to the bridging veins and superficial cerebral vessels caused by a sudden change in intracranial pressure, combined with brain fragility and a prolonged PT-INR resulting from long-term warfarin therapy for secondary stroke prevention. According to the ESC guidelines, an INR below 2.0 is considered acceptable for minor bleeding events [3]. Usually, contralateral bleeding does not occur in surgery for CSDH, so we classified it as minor bleeding and performed the surgery without reversing warfarin. Although the INR was 1.7 at the time of surgery and within the range generally considered acceptable for minor procedures, the coexistence of anticoagulation and antiplatelet therapy likely increased the risk of postoperative bleeding. However, in a patient at risk, as in this case, reversal of warfarin would have been necessary. Moreover, a careful follow-up is necessary to detect possible complications. This case underscores the importance of recognizing how multiple systemic and local factors can interact to produce postoperative hemorrhagic complications, and it may help inform strategies to minimize such risks in similarly complex patients.

## Conclusions

In conclusion, contralateral ASDH following burr-hole surgery is a rare but clinically relevant complication that may be associated with brain shift and coagulation-related factors. The present case highlights the importance of carefully balancing the risks of anticoagulation reversal and thromboembolic events, rather than suggesting a uniform management strategy. Vigilant postoperative monitoring and timely imaging are essential to detect remote hemorrhagic complications early. The patient in this case achieved a full neurological recovery, but this favorable outcome should be interpreted within the context of a single case.
